# Diagnostic, predictive and prognostic molecular biomarkers in clear cell renal cell carcinoma: A retrospective study

**DOI:** 10.1002/cnr2.2116

**Published:** 2024-06-04

**Authors:** Jian Deng, ShengYuan Tu, Lin Li, GangLi Li, YinHui Zhang

**Affiliations:** ^1^ Department of Oncology Hejiang Hospital of Traditional Chinese Medicine Luzhou People's Republic of China; ^2^ School of Basic Medical Sciences Southwest Medical University Luzhou People's Republic of China; ^3^ School of Stomatology Southwest Medical University Luzhou People's Republic of China; ^4^ Department of Pharmacy The Affiliated Hospital of Southwest Medical University Luzhou People's Republic of China; ^5^ Department of Anesthesiology Hospital (T.C.M) Affiliated to Southwest Medical University Luzhou People's Republic of China; ^6^ Department of Pharmacy Hejiang Hospital of Traditional Chinese Medicine Luzhou People's Republic of China

**Keywords:** biomarkers, clear cell renal cell carcinoma, diagnostic markers, predictive markers, prognostic markers

## Abstract

Clear cell renal cell carcinoma (ccRCC) is a common and aggressive subtype of kidney cancer. Many patients are diagnosed at advanced stages, making early detection crucial. Unfortunately, there are currently no noninvasive tests for ccRCC, emphasizing the need for new biomarkers. Additionally, ccRCC often develops resistance to treatments like radiotherapy and chemotherapy. Identifying biomarkers that predict treatment outcomes is vital for personalized care. The integration of artificial intelligence (AI), multi‐omics analysis, and computational biology holds promise in bolstering detection precision and resilience, opening avenues for future investigations. The amalgamation of radiogenomics and biomaterial‐basedimmunomodulation signifies a revolutionary breakthrough in diagnostic medicine. This review summarizes existing literature and highlights emerging biomarkers that enhance diagnostic, predictive, and prognostic capabilities for ccRCC, setting the stage for future clinical research.

## 1 INTRODUCTION

Renal cell carcinoma (RCC), originating in the epithelium of kidney tubules, stands out as a prevalent malignant tumor within the urinary system, accounting for around 3% of adult malignancies.^1^ Among RCCs, clear cell renal cell carcinoma (ccRCC) emerges as the predominant and aggressive histological subtype, encompassing 75%–85% of all RCC cases.^2^ Over the past two decades, there has been a twofold increase in the global incidence of ccRCC, accompanied by a yearly 1% rise in mortality rates.^3^

Following early diagnosis and effective surgical intervention, patients can attain a 5 year survival rate of up to 93%.^4^ Regrettably, the lack of typical early clinical symptoms leads to the diagnosis of nearly 40% of ccRCC patients during the advanced stages of the disease, when tumors are already sizable and/or have metastasized. At this juncture, offering effective therapeutic strategies becomes challenging, resulting in an unfavorable prognosis, with a 5 year survival rate of below 20%.^5^, ^6^ Biomarkers stand as pivotal tools within cancer research, particularly for ccRCC. These indicators are extensively present in blood and urine, enabling the facile collection of specimens and offering a minimally invasive or noninvasive, convenient, sensitive, and efficient means of monitoring.^7^ They wield substantial significance in the early diagnosis of ccRCC, identifying patients at heightened risk of disease recurrence or progression,^8^ and augmenting patient survival. Beyond their role in elucidating cancer diagnosis and determining patient prognosis, biomarkers also serve as predictive factors for anticipating patient responses to therapies or interventions.^9^

Given the distinct sensitivities of patients with renal clear cell carcinoma to targeted or immunotherapy drugs, instances of drug resistance are frequent (primary and secondary drug resistance). Furthermore, the efficacy of radiotherapy and chemotherapy remains suboptimal. Thus, the identification of biomarkers to forecast the therapeutic effectiveness of drugs and to assess patient prognoses assumes paramount importance. This endeavor is vital for devising personalized treatment plans.^10^ Enhancing the early diagnostic rate of ccRCC, mitigating metastasis in RCC, and elevating survival rates for advanced patients are pressing objectives. Thus, urgent efforts are needed to explore early diagnostic markers of ccRCC.^11^ Equally important are the identification and development of molecular biomarkers linked to drug treatment sensitivity, enabling the prediction of therapeutic outcomes for renal clear cell carcinoma and prognosis assessment.^11^

**FIGURE 1:**
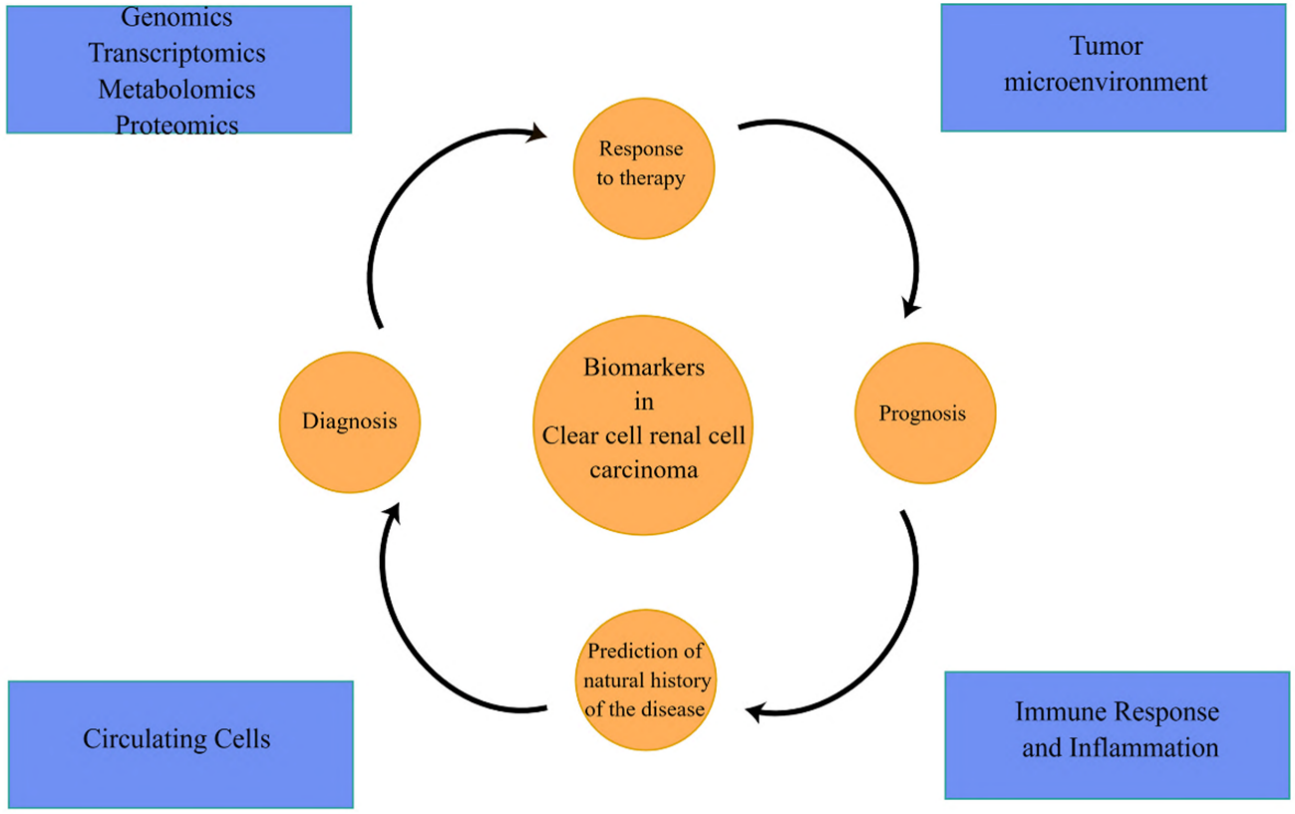
Illustration of the spectrum and variability of biomarkers involved in the diagnosis, prognosis, and prediction of clear cell renal cell carcinoma.

Within this article, we have comprehensively outlined established biomarkers, along with potential ones, and elucidated new research avenues focused on diagnosing, predicting, and prognosticating renal clear cell carcinoma. This compilation serves as a cornerstone for forthcoming clinical investigations (Figure 1 and Table 1).

**TABLE 1:** Summary of the main roles of commonly used biomarkers.

Biomarker	Diagnostic	Prognostic	Predictive	Clinical Specimen	References
ccRCC1‐4	×	×		ccRCC tissue	12, 13
TLR3	×	×		ccRCC tissue	14, 15
IL‐6/IL‐8	×			ccRCC tissue and serum	16, 17, 18, 19, 20, 21
PD‐L1	×			Plasma, peripheral blood, and ccRCC tissue	22, 23, 24, 25, 26, 27, 28
BIRC5/survivin	×			ccRCC tissue	29, 30, 31
Immune Response and Inflammatory Markers	×			Plasma	32, 33
NNMT			×	ccRCC tissue, plasma, and kidney tissues	34, 35, 36, 37
Exosomes				Urine, blood, and ccRCC tissue	38, 39
DNA Methylation			×	kidney tissues, urine	40, 41, 42, 43, 44, 45, 46, 47
CAIX/CA9			×	Serum and ccRCC tissue	48, 49, 50, 51, 52
CTCs			×	Blood and peripheral blood	53, 54
3p					55, 56, 57, 58, 59, 60, 61, 62, 63, 64, 65, 66, 67, 68
microRNAs				Serum, plasma, and urine	69, 70, 71, 72, 73, 74, 75, 76, 77, 78, 79, 80, 81, 82, 83, 84

## 2 BIOMARKERS

### 2.1 Predictive role

#### 2.1.1 ccRCC1‐4

An analysis of The Cancer Genome Atlas (TCGA) dataset identified four distinct molecular subgroups within ccRCC, designated as m1–m4.^12^ Prognostically, ccRCC1 and ccRCC4 tumors exhibited a lower response rate, reduced overall survival (OS), and shorter progression‐free survival (PFS) compared to ccRCC2 and ccRCC3 tumors. Through unsupervised transcriptome analysis, four robust ccRCC subtypes (ccRCC1–ccRCC4) were discerned, each demonstrating varying responses to sunitinib treatment. Specifically, ccRCC2 and ccRCC3 tumors displayed significantly improved median OS (*p* = .0003) and PFS (*p* = .0001) compared with ccRCC1 and ccRCC4 tumors.^13^ These molecular subtypes emerged as the only significant covariates in a multivariate Cox regression model for both PFS and OS. Collectively, these findings suggest that molecular subtypes of ccRCC serve as predictive markers for sunitinib treatment response in patients with metastatic disease. In this context, it is important to note that the ccrcc1‐4 molecular subtyping, proved to be related to older therapies.^85^

#### 2.1.2 TLR3

TLR3 (toll‐like receptor 3) is a critical component of the innate immune system, responsible for mediating antiviral responses through the recognition of viral elements such as double‐stranded RNA and polyinosinic–polycytidylic acid poly (I:C).^14^ It also induces the production of type I interferons, particularly IFN‐λ. Previous studies have shown that TLR3 is abundantly expressed in ccRCC tumors.^15^ In a sub‐analysis of the AXIS trial, which compared the efficacy of axitinib and sorafenib in ccRCC patients, it was observed that higher expression levels of TLR3 (≥ median) in axitinib‐treated patients were associated with longer PFS (HR: 0.4, 95% CI: 0.2–0.9; *p* = .023). Conversely, lower expression levels of TLR3 (< median) in sorafenib‐treated patients correlated with both longer PFS (HR: 3.9, 95% CI: 1.4–10.7; *p* = .005) and OS (HR: 3.0, 95% CI: 1.1–8.0; *p* = .022).^86^

### 2.2 Prognostic and predictive role

#### 2.2.1 IL‐6/IL‐8

Interleukin‐6 (IL‐6) and interleukin‐8 (IL‐8) have been identified as significant molecular events in the late stages of ccRCC pathogenesis. These cytokines have been suggested as both predictive and prognostic biomarkers for patients diagnosed with ccRCC.^16^, ^17^, ^18^, ^19^ Huang et al. demonstrated that resistance to sunitinib, a tyrosine kinase inhibitor (TKIs), is mediated through a mechanism that circumvents antiangiogenic effects. Specifically, they observed that neovascularization could potentially be reactivated via a VEGF/VEGFR‐independent pathway.^16^ In their study, 89 factors were screened, revealing elevated plasma levels of IL‐8—a potent proangiogenic chemokine—in mice with sunitinib‐resistant tumors compared to those with sunitinib‐sensitive tumors.

In a separate study, Petillo et al. elucidated that the activation of the transcription factor STAT3 by IL‐6 promotes angiogenesis. This is achieved through the induction of vascular endothelial growth factor (VEGF) and fibroblast growth factor (FGF) expression in tumor cells.^16^

Patients exhibiting elevated baseline serum levels of IL‐6 and IL‐8 are correlated with reduced PFS and/or OS when treated with pazopanib or sunitinib.^19^, ^20^, ^21^ Studies by Huang et al. showed that increased plasma levels of IL‐8 were detected in the plasma of mice with sunitinib‐resistant tumors compared to mice bearing sunitinib‐responsive tumors and treatment with IL‐8 neutralizing antibodies reinstated sensitivity to sunitinib; Huang et al. further substantiated this by revealing that mice with sunitinib‐resistant tumors exhibited increased plasma levels of IL‐8 compared to those with sunitinib‐responsive tumors. Importantly, the administration of IL‐8 neutralizing antibodies restored sunitinib sensitivity in these resistant models.^16^

#### 2.2.2 PD‐L1

PD‐L1, a ligand for programmed death‐1 (PD‐1), plays a crucial role in inhibiting T‐cell‐mediated immune responses. As an immunosuppressive molecule, PD‐L1 attenuates the activation of T cells, thereby facilitating tumor progression. In clear ccRCC, PD‐L1 protein is present in 10%–25% of tumor cells and has been identified as a biomarker indicative of a poorer prognosis.^22^ A meta‐analysis further supports the utility of PD‐L1 expression as a valuable prognostic tool in ccRCC patients.^23^ Montemagno et al. reported that the levels of sPD‐L1 (soluble forms) and sPD‐1 (soluble forms) were correlated to clinical parameters and PFS. High levels of sPD‐1 or sPDL1 were not correlated to PFS under bevacizumab while they were independent prognostic factors of PFS in the sunitinib group.^24^

In a complementary study by Larrinaga et al. the authors advocate for the clinical utility of a sPD‐L1 level exceeding 793 ng/mL as an independent and novel prognostic indicator for ccRCC patients.^25^ They also observed that sPD‐L1 levels increased across International Metastatic Renal Cell Carcinoma Database Consortium (IMDC) prognostic groups among patients with metastatic ccRCC. This elevation was further correlated with the clinical response of these patients to systemic therapy.

Intriguingly, a multivariate analysis revealed that high expression levels of AXL in conjunction with PD‐L1 were associated with a trend toward reduced OS, with a hazard ratio (HR) of 2.01 (95% CI, 1.18–3.44, *p* = .084).^26^ These findings suggest AXL as a potential factor contributing to resistance against PD‐1 blockade. They provide strong evidence supporting the screening of both AXL and PD‐L1 expression for the effective management of advanced ccRCC. Additionally, the co‐expression of HHLA2 and PD‐L1 was found to have a detrimental impact on the prognosis of ccRCC patients.^27^

Lastly, in the context of localized ccRCC, the presence of infiltrating CD8 + PD‐1 + Tim‐3 + Lag‐3+ exhausted tumor‐infiltrating lymphocytes (TILs) alongside ICOS Treg cells has been associated with an unfavorable prognosis for patients one. This subset of patients could potentially derive benefit from adjuvant therapy involving agents that modify the tumor microenvironment (TME) and employ checkpoint blockade strategies.^28^

#### 2.2.3 BIRC5/Survivin

The BIRC5 gene, a member of the inhibitor of apoptosis (IAP) family, encodes the Survivin protein. Prior in vitro studies have established that Survivin expression serves as an independent predictor for the progression and mortality associated with ccRCC.^29^ This suggests that Survivin may offer a novel target for the development of adjuvant therapies. In a separate in vitro study, Linehan et al. demonstrated that the co‐expression of Survivin and B7‐H1, a ubiquitous anti‐apoptotic receptor, offers an enhanced predictive value for assessing ccRCC tumor aggressiveness.^30^

Moreover, Krambeck et al. conducted an extensive analysis of 1,994 ccRCC specimens and reported that patients with high Survivin (Survivin^Hi^) and positive B7‐H1 (B7‐H1^+^) expression in their ccRCC tumors are at an elevated risk of mortality due to the disease.^31^ The prognostic and clinicopathological significance of Survivin expression in determining renal cancer patient outcomes has thus been further corroborated.

#### 2.2.4 Immune response and inflammatory markers

RCC is one of the most immune‐infiltrated tumors.^87^, ^88^, ^89^ Emerging evidence suggests that the activation of specific metabolic pathway have a role in regulating angiogenesis and inflammatory signatures.^90^, ^91^ Features of the TME heavily affect disease biology and may affect responses to systemic therapy.^92^, ^93^, ^94^, ^95^

Inflammation is an important component of both the progression of carcinogenesis and antitumor response. O'Brian et al. reported that the C‐reactive protein and the neutrophil/lymphocyte (NLR) ratio are independent prognostic factors of poor survival in patients with ccRCC. High CRP is also associated with numerous poor prognostic indicators including larger tumor size, higher grade and stage, lymphatic involvement, microvascular invasion, and aggressive histopathological findings such as spindle morphology and sarcomatoid morphology.^32^ In a study by Eduard Roussel, elevated baseline CRP and NLR predict worse OS and PFS on nivolumab in m‐ccRCC patients. Including baseline CRP in the IMDC pro gnostic model improves its discriminatory power to predict OS and PFS since the start of nivolumab.

Similarly, in another study by Yano et al., OS for patients with CRP >1 mg/dL was significantly shorter than those with CRP <1 mg/dL in both ccRCC (58 patients: *p* = .009) and nccRCC (16 patients: *p* = .008).^33^

### 2.3 Diagnostic and prognostic role

#### 2.3.1 NNMT

Nicotinamide N‐methyltransferase (NNMT) is responsible for catalyzing the N‐methylation of nicotinamide, utilizing S‐adenosyl‐L‐methionine as the methyl donor. Notably, NNMT has been assessed both as a diagnostic marker and a prognostic indicator for survival in patients diagnosed with ccRCC.^34^ A comprehensive proteotranscriptomic analysis unveiled NNMT as a dependable biomarker for detecting ccRCC2, particularly in advanced‐stage cases.^35^ In RCC, NNMT protein expression was significantly heightened, particularly within clear cell RCC (82.8%).^36^

Through univariate survival analysis, it was established that heightened levels of NNMT enzyme activity correlated with an unfavorable prognosis. This notion was reinforced by Kaplan–Meier curves, which illustrated prolonged survival periods among patients with tumors showcasing low NNMT expression, whereas the opposite was observed for those with high NNMT expression.^37^

#### 2.3.2 Exosomes

Exosomes, extracellular vesicles stemming from cells, exhibit diameters ranging from 40 to 160 nm. These vesicles harbor an assortment of biomarkers, encompassing disease‐specific DNA, RNA, metabolites, signaling molecules, and cell‐surface proteins.^38^ Remarkably, cancer cells excrete exosomes at a rate 10 times greater than that of normal cells. Scrutiny of exosomes derived from tumors yields pivotal insights into tumor‐related profiles.^39^

Investigations into exosomal miRNA as a plausible ccRCC biomarker have revealed the communicative role of miRNA in intercellular processes.^96^ Employing next‐generation sequencing (NGS), it was uncovered that exosomal miR‐30c‐5p holds promise as a diagnostic biomarker for early‐stage ccRCC. Furthermore, this miRNA may modulate the expression of HSPA5, a protein linked to ccRCC progression.^97^ Notably, miR‐126‐3p, when combined with miR‐449a or miR‐34b‐5p, exhibits significant potential in distinguishing ccRCC patients from healthy individuals. The diagnostic prowess of this combination was evident (miR‐126‐3p‐miR‐449a: area under the curve AUC = 0.84; 95% confidence interval CI: 0.7620–0.9151; *p* < .001; miR‐126‐3p‐miR‐34b‐5p: AUC = 0.79; 95% CI: 0.7013–0.8815; *p* < .001).^96^ Notably, exosomes from cancer stem cells (CSCs) introduce bioactive miR‐19b‐3p of epithelial‐mesenchymal transition (EMT) by inhibiting PTEN expression in cancer cells through CSC exosomes. CD103 facilitates the targeting of CSC exosomes to cancer cells and organs, thereby conferring enhanced lung metastatic capacity to ccRCC, and highlighting CD103 exosomes as potential diagnostic indicators of metastatic potential.^98^

In ccRCC patients, Tsuruda et al. observed a significant elevation in the protein expression levels of RAB27B within sunitinib‐resistant ccRCC cell lines. As a central participant in exosome secretion, RAB27B may play a role in drug resistance through the MAPK and VEGF signaling pathways.^99^

Moreover, exosome‐associated long noncoding RNAs (lncRNAs) have emerged as pertinent to anti‐tumor immunity and ccRCC prognosis. Through the deployment of Bayesian spike‐and‐slab lasso regression, a prognostic model was constructed, exhibiting enhanced reliability in predicting 1, 3, and 5 year survival. Consequently, Kaplan–Meier curves unveiled a bleaker prognosis in the high‐risk score cohort (*p* < .001).^100^

#### 2.3.3 DNA methylation patterns

DNA methylation is a process orchestrated by DNA methyltransferase (DNMT) enzymes, leading to the alteration of cytosine residues through the addition of a methyl side group, resulting in the formation of 5‐methylcytosine.^40^ This phenomenon is a significant component of the molecular machinery governing cancer evolution and prognosis. DNA methylation fosters the progression of malignant cells by activating oncogenes and deactivating tumor suppressor genes.^41^ For over two and a half decades, the hypermethylation of CpG islands, which leads to the suppression of tumor suppressor gene expression, has been extensively studied in the context of ccRCC.^42^ An independent validation of a set of 11 CpG sites within The TCGA data have demonstrated an association with roughly 90% or higher sensitivity and 80% specificity in distinguishing between kidney cancer and normal tissues or ccRCC.^43^

In a similar vein, a panel composed of ZNF677, FBN2, PCDH8, TFAP2B, and TAC1 has been linked to approximately 82% sensitivity and 96% specificity. Remarkably, the hypermethylation of ZNF677 and PCDH8 within tissue samples exhibited significant associations with various unfavorable clinicopathological parameters. For the purpose of detecting ccRCC through urine samples, the highest diagnostic accuracy was observed with a panel involving ZNF677 and PCDH8 (with or without FBN2 or FLRT2), yielding sensitivities ranging from 69% to 78% and specificities from 69% to 80%.^44^ Zhou et al. introduced a novel prognostic model based on six methylation‐driven genes (SAA1, FUT6, SPATA18, SHROOM3, AJAP1, and NPEPL1) that enables the prediction of OS in individuals diagnosed with ccRCC. Moreover, they identified enhanced drug sensitivity to sorafenib, axitinib, gefitinib, erlotinib, cediranib, ZM447439, RO‐3306, and cytarabine within the low‐risk group, whereas the high‐risk group exhibited greater sensitivity to cisplatin and camptothecin.^45^ Additionally, promoter methylation of HOXA9 and OXR1 was observed in 73% and 87% of renal cell tumors, respectively. A two‐gene methylation panel consisting of OXR1 and MST1R demonstrated the ability to identify ccRCC with an impressive 90% sensitivity and 98% specificity.^46^

An analysis of promoter‐methylated genes within ccRCC patients unveiled a notable reduction in survival among patients with more than 12 hypermethylated genes compared with those with fewer than 12 hypermethylated genes.^47^

#### 2.3.4 Carbonic anhydrase IX (CAIX/CA9)

Carbonic anhydrase IX (CAIX), one of the 14 isoforms of carbonic anhydrase enzymes present in humans, is found to be significantly upregulated in various cancer types. In RCC, CAIX stands out as a well‐characterized enzyme. Its expression is modulated by hypoxia‐inducible factor 1 alpha (HIF‐1α), and it is known to impact processes related to hypoxia. As part of the histological subtype diagnosis, immunohistochemistry analyses play a pivotal role. These analyses primarily rely on three immunomarkers: cytokeratin 7 (CK7), α‐methyl acyl‐CoA racemase (AMACR), and CAIX. Together, these markers form an initial panel for diagnosis.^48^ Zhu et al. discovered that CAIX aptamer‐functionalized targeted nanobubbles have the potential to enhance ultrasound molecular imaging of tumor cells, thereby elevating the accuracy of early diagnosis.^49^ Similarly, Minn et al. synthesized 64Cu XYIMSR‐06, a dual‐motif CAIX ligand, which has demonstrated the capability to visualize ccRCC using positron emission tomography.^50^ This suggests that molecular imaging agents targeting CAIX hold promise for enhancing diagnostic precision.

In the ccRCC patient cohort as a whole, the Kaplan–Meier survival analysis demonstrated a notable correlation between elevated serum levels of carbonic anhydrase IX (CAIX) and abbreviated OS (HR = 2.65, 95% CI = 1.19–5.92, *p* = .0136). Similarly, within the major subgroup of patients subjected to temsirolimus and bevacizumab treatments, the Kaplan–Meier survival curve showcased a pronounced link between elevated serum CAIX levels and diminished OS (*p* = .0006). Notably, diminished CAIX staining (≤85% staining) emerged as an independent adverse prognostic factor for survival.^51^ However, in contrast, Büscheck et al. conducted a retrospective examination of 1809 cases of RCC, adhering to the 2016 World Health Organization (WHO) classification criteria. Their investigation revealed a noteworthy association wherein heightened CAIX expression within the ccRCC group corresponded with lower Fuhrman grade and ISUP classification, decreased tumor stage, and the absence of distant metastases. Furthermore, this heightened CAIX expression was aligned with improved recurrence‐free survival and OS outcomes.^52^

#### 2.3.5 Circulating tumor cells

Circulating tumor cells (CTCs) have been explored as both diagnostic tools and prognostic indicators of survival in patients diagnosed with ccRCC.^53^, ^54^ However, isolating CTCs poses a challenging endeavor, and previous methodologies to assess CTCs in the context of ccRCC have yielded limited clinical utility due to either low detection rates or elevated false positives.^53^ Nonetheless, a breakthrough emerged when Bade et al. identified renal‐specific markers expressed by ccRCC CTCs through the application of flow cytometry. They further harnessed a remarkably sensitive CTC microfluidic platform to validate the presence of distinct subsets of CTCs exhibiting diverse combinations of cytokeratin (CK), epithelial cell adhesion molecule (EpCAM), CAIX, and/or carbonic anhydrase XII (CAXII). The utilization of receiver operating characteristic (ROC) curve analysis for two CTC groups (CAXII S+ vs. CK+ either CK S+ or dual‐positive (DP)) revealed a significant correlation between the number of CK+ CTCs (with an optimal cutoff of 2.6/mL) and radiographic progression (*p* < .05). In contrast, the number of CAXII S+ CTCs did not exhibit a significant correlation (*p* > .05). This finding underscores the significance of acknowledging CTC heterogeneity in the context of ccRCC CTC diagnostics.^53^ Furthermore, an immunohistochemistry analysis unveiled a heightened likelihood of detecting CTCs with an increase in tumor size, particularly in cases of ccRCC.^54^

### 2.4 Diagnostic, prognostic, and predictive role

#### 2.4.1 Genes on chromosome 3p

The loss of chromosome 3p and inactivating mutations within the tumor suppressor genes situated on chromosome 3p constitute pivotal oncogenic driver events in ccRCC. Evidently, over 90% of sporadic ccRCC cases exhibit a deletion of chromosome 3p, potentially leading to the loss of heterozygosity concerning the tumor suppressor genes located on this chromosome. Predominantly affected are VHL, PBRM1, BAP1, and SETD2 genes. Hence, chromosome 3p alterations‐based fluorescence in situ hybridization (FISH) emerges as a suitable diagnostic tool for renal mass assessment. Furthermore, the four mentioned tumor suppressor genes demonstrate a high frequency of mutations within the ccRCC context.^55^, ^56^ Among these genes, the Von Hippel–Lindau (VHL) tumor suppressor gene stands out as the most commonly mutated gene in ccRCC pathogenesis. Central to its function is the orchestration of ubiquitylation and proteasomal degradation of hypoxia‐inducible factor (HIF). Disruption of VHL through deletion or inactivating mutations culminates in HIF accumulation and subsequent elevation of downstream target genes, including erythropoietin (EPO), VEGF, glucose transporter‐1 (GLUT‐1), and transforming growth factor‐α (TGF‐α). This cascade bears immense significance for cellular proliferation, angiogenesis, and tumorigenesis.^57^, ^58^, ^59^, ^60^ Recent investigations into the VHL–HIF pathway's oncogenic mechanism have catalyzed the advancement of targeted therapies for ccRCC. The emergence of TKIs like Belzutifan and monoclonal antibodies such as bevacizumab has significantly enhanced patient survival and prognosis.^59^, ^60^, ^61^, ^62^ Despite VHL's critical role in the molecular pathogenesis of ccRCC, its exact prognostic and predictive implications remain uncertain. A recent meta‐analysis, however, suggests that VHL gene alterations appear to lack prognostic and predictive value for patients with ccRCC.^63^

PBRM1, BAP1, and SETD2 are additional genes functioning as epigenetic regulators, instrumental in the orchestration of gene transcription and commonly implicated in chromosome remodeling. PBRM1, standing as the second most frequently mutated gene in ccRCC, appears altered in approximately 45% of ccRCC cases and is often co‐deleted alongside the VHL gene.^58^ Notably, PBRM1 has emerged as a noteworthy prognostic and predictive marker for early‐stage ccRCC. Diminished expression of PBRM1 has been significantly linked to unfavorable clinical outcomes, amplifying the likelihood of reduced patient tolerance and inferior prognosis in response to immune checkpoint inhibitors.^64^, ^65^ Within the same context, BAP1 and SETD2, also situated on chromosome 3p, register mutations in 10%–15% of ccRCC patients each, with both mutations correlating with adverse patient prognosis.^58^ Tumors harboring BAP1 mutations exhibit heightened Fuhrman grading and mTORC1 activation status, enabling the prediction of patient response to mTORC1 inhibitors. This underscores the favorable prognostic and predictive value of BAP1.^66^, ^67^ Furthermore, SETD2 mutations and reduced expression are associated with compromised cancer‐specific survival. These alterations elevate the risk of disease recurrence or metastasis, imparting additional impetus for their prognostic relevance.^68^

#### 2.4.2 microRNAs

MicroRNAs (miRNAs/miRs) constitute a class of small endogenous noncoding RNAs, spanning 19–22 nucleotides, that proficiently modulate gene expression through translation repression and acceleration of recognized messenger RNA (mRNA) degradation. This regulatory action transpires via target recognition at the 3′ untranslated region (UTR) of the intended gene, ultimately governing protein expression.^69^, ^70^ This distinctive functionality has spurred their detection within diverse bodily fluids, encompassing saliva, serum, breast milk/colostrum, urine, and peritoneal cavity fluid. Significantly, miRNAs have recently ascended as prospective cancer biomarkers.^70^, ^71^

Winter et al. substantiated that ccRCC tissue boasts a distinct miRNA expression profile, enabling its differentiation from normal tissue.^72^ Additionally, Shu et al. accomplished a multi‐phase study design, culminating in the identification of a 17‐miRNA signature capable of accurately discerning over 95% of tumor/adjacent samples. This remarkable precision arises from the inherent stability and functionality of the identified miRNA panel. Furthermore, within the same investigation, miR‐204‐5p and miR‐139‐5p were identified as linked to poor survival outcomes.^73^

Liang et al. contributed a panel of three miRNAs, namely miR‐21, miR‐584, and miR‐155, manifesting noteworthy diagnostic performance in ccRCC. This panel exhibited both high sensitivity (98.3%) and specificity (97.2%), and emerged as an independent prognostic factor for OS, substantiated by both univariate and multivariate Cox regression analyses.

Several studies have also furnished compelling evidence attesting to the prognostic potential of miRNAs. For instance, heightened expression of miR‐630, miR‐210‐3p, miR‐210, miR‐141, and miR‐146a‐5p has consistently been linked to the prediction of adverse prognosis in ccRCC patients.^74^, ^75^, ^76^, ^77^, ^78^ In contrast, downregulation of miR‐30a‐5p, miR‐194, miR‐217, miR‐129‐3p, miR‐178, miR‐204‐5p, miR‐139‐5p, and miR‐124‐3p has been correlated with inferior survival outcomes.^79^

However, MicroRNAs as biomarkers of ccRCC have been recently reviewed in some literatures.^101^, ^102^ It is necessary to conduct large clinical trials in the future to verify whether these tools are effective in clinical decision‐making.

## 3 METABOLOMICS

RCC is essentially a metabolic disease characterized by a reprogramming of energetic metabolism.^103^, ^104^, ^105^, ^106^, ^107^ In particular the metabolic flux through glycolysis is partitioned,^108^, ^109^, ^110^ and mitochondrial bioenergetics and OxPhox are impaired, as well as lipid metabolism.^111^, ^112^, ^113^ These studies explored many metabolic biomarkers with diagnostic and prognostic role in ccRCC.

Similarly, ccRCC has emerged as a metabolic disease, with metabolic reprogramming recently acknowledged as a pivotal and prominent hallmark. This reprogramming is orchestrated by the loss of tumor suppressor genes and activation of oncogenes.^114^ Gene mutations involved in this process lead to substantial metabolic alterations, encompassing glucose, lipid, and amino acid metabolism. Notably, cancer cells meet their augmented energy and building block requirements by reshaping their metabolism, effectively fueling tumor growth.^115^ A prime example is the Warburg effect, a hallmark of metabolic reprogramming in cancer cells that furnishes the requisite energy for tumor proliferation and fosters a lactate‐enriched milieu pivotal for tumor progression.^116^ In this context, emerging evidence underscores the prognostic potential of lactate‐related genes, including HADH, FBP1, and TYMP.^117^ Crucial to tumor cellular energy metabolism, glucose metabolism undergoes essential adaptations in ccRCC to ensure ample energy generation. Recent investigations highlight HK2, among the upregulated key enzymes in this process, as associated with immune cell infiltration and prognostic indicators in kidney cancer patients.^118^ Furthermore, the formation of lipid droplets and dysregulated lipid metabolism constitute key features of ccRCC. Dysregulation of lipid metabolism, driven by lipogenic genes, correlates with inferior clinical outcomes in ccRCC patients.^119^ Notably, adipokines and lipid species have emerged as potential diagnostic and treatment monitoring biomarkers, and targeting fatty acid metabolism holds promise as a therapeutic strategy for ccRCC.^120^ Amino acid metabolism, including glutamine, tryptophan, and arginine pathways, significantly influences tumor progression. Critical enzymes in the arginine pathway, argininosuccinate lyase (ASL) and argininosuccinate synthase‐1 (ASS1), are essential in this context. The downregulation of these enzymes correlates with chemotherapy drug resistance and adverse prognosis.^121^ In summary, advances in tumor metabolomics have unveiled these modifications or reprogramming of metabolic pathways, laying the foundation for early and effective tumor diagnostic methods, identification of potential biomarkers, and the exploration of novel therapeutic targets and strategies.

## 4 NOVEL CELL DEATH MECHANISMS

Cell death serves as a physiological or pathological mechanism in multicellular organisms for the elimination of superfluous or detrimental cells. This process is crucial for the development and homeostasis of multicellular organisms, as it facilitates normal cellular renewal and sustains the structural and functional integrity of various tissues. Traditionally, apoptosis was considered the primary form of regulated cell death. However, emerging research over recent decades has identified multiple forms of regulated necrosis, such as ferroptosis, cuproptosis, and pyroptosis, which have implications in pathological conditions like cancer and inflammation. These discoveries have paved the way for new therapeutic interventions targeting regulated, nonapoptotic cell death pathways.^122^ Here, we describe the regulatory pathways of ferroptosis, cuproptosis, and pyroptosis.

Ferroptosis is a recently identified form of cell death characterized by the accumulation of iron‐dependent lipid peroxides, primarily induced by iron overload and the generation of reactive oxygen species (ROS)‐dependent lipid peroxides. Notably, RCC tissues exhibit elevated iron levels compared to solid tumors in other organs, including the liver, prostate, and stomach. Furthermore, lower intratumoral iron concentrations and elevated renal epithelial iron levels are associated with significantly reduced metastasis‐free survival in ccRCC patients.^123^ Consequently, the modulation of iron metabolism and the subsequent induction of ferroptosis may serve as promising therapeutic strategies for mitigating the progression of RCC.

Cuproptosis is another recently identified form of cell death, characterized by an imbalance in copper ion homeostasis. This process is mediated through the direct interaction between copper ions and the fatty acid components of the tricarboxylic acid cycle (TCA cycle), resulting in the aggregation of acylated proteins and the subsequent depletion of ferritin. This cascade of events culminates in protein toxicity stress, ultimately leading to cell death. In ccRCC, two distinct subtypes based on copper poisoning scores—CPCS1 (high score) and CPCS2 (low score)—exhibit unique clinical and biological characteristics. Notably, the CPCS2 subtype is associated with a more advanced clinical stage and poorer prognosis, potentially due to its role in regulating keratinization and epidermal cell differentiation, thereby accelerating cancer progression.^124^

Pyroptosis is another form of programmed cell death, distinct for its reliance on inflammasome‐dependent mechanisms mediated by the GASD family of proteins. This process occurs more rapidly than apoptosis and is typically accompanied by the release of a plethora of pro‐inflammatory cytokines. These cytokines, such as IL‐1 β and IL‐18, are released through plasma membrane pores formed by caspase‐1 activation, leading to cell lysis. In a seminal clinical study, Fu et al. systematically investigated the transcriptional variations and expression patterns of necroptosis‐ and pyroptosis‐related genes (NPRGs). Utilizing a necrotic pyroptosis gene (NPG) score, they constructed and validated a prognostic model, providing the first comprehensive evidence for a significant interplay between the TME defined by necrotic pyroptosis and the prognostic outcomes in ccRCC.^125^

## 5 CANCER STEM CELLS

CSCs are a subset of tumor cells endowed with stem cell‐like properties, offering significant therapeutic targets and foundational research avenues for the treatment of various malignancies. They hold the potential for achieving complete tumor eradication. However, research on renal CSCs has been relatively nascent compared to investigations into stem cells within other urological malignancies.

The expression of some stem cell markers in different RCC cell lines is a controversial issue.^126^, ^127^ For example, Galleggiante et al. described and characterized a population of resident CD133+/CD24+ cancer cells in patients with clear cell RCC.^128^ In addition Varna et al. identified a larger number of CD133/CXCR4–co‐expressing cells in perinecrotic versus perivascular areas in RCC tissue.^129^ The use of CD105 as a renal CSC marker was questioned in many studies that showed how other putative subpopulations of cells with CSC‐like properties are CD105.^130^, ^131^ Moreover, Canis et al showed that stable transfection of CD133 in the human embryonic kidney 293 (HEK293) cell line induced tumor‐initiating properties in these cells. In addition, HEK293 CD133high transfectants, when injected into SCID (Severe combined immunodeficiency) mice, generated tumors with at least a 1000‐fold increased frequency as compared with CD133low cells.^132^ CD133 and CXCR4 have been proposed as potential markers for identifying renal CSCs.^133^ The utility of these markers as specific identifiers for renal CSCs awaits validation through further experimental studies.

## 6 CHALLENGES AND FUTURE DIRECTIONS

Despite significant advancements in technology, the underlying biology and nature of ccRCC remain incompletely understood by both clinicians and researchers. One of the primary challenges lies in the identification of reliable biomarkers for early diagnosis. Although a plethora of potential proteomic markers has been identified, only a few have demonstrated robust scientific and clinical utility. The low prevalence of ccRCC, coupled with the lack of specific early‐stage symptoms that could prompt diagnostic testing, makes the identification of an ideal surrogate marker for population‐wide screening particularly challenging.

Additionally, ccRCC is characterized by high malignancy and resistance to conventional chemotherapy and radiotherapy.^134^ Emerging approaches, such as artificial intelligence and multi‐omics analyses, in conjunction with computational biology, offer the potential for enhanced detection accuracy and robustness. Radiogenomics, in particular, represents a paradigm shift in diagnostic medicine, showing promise in small‐scale retrospective studies. However, further research is imperative for the identification and validation of biomarkers prior to their integration into clinical practice.^135^ Momin et al. observed that the attachment of cytokines to the collagen‐binding protein lumican resulted in a reduction of systemic toxicity and side effects, coupled with an extension of local retention.^136^ Cytokine collagen anchoring represents a straightforward and tumor‐agnostic approach to augment systemic immunotherapy safely, particularly when its efficacy might otherwise be limited. The utilization of biomaterials to enhance immune modulation through this strategy is a burgeoning area of interest.^137^ Nevertheless, additional research is also imperative to explore and validate its clinical applications.

## 7 CONCLUSIONS

ccRCC represents an escalating global public health concern.^138^ Inaccurate test results contribute to delayed diagnoses and, consequently, poorer prognoses. Although various pharmaceutical agents are available for the clinical management of ccRCC, the majority lack high sensitivity and specificity, rendering current screening methods suboptimal. There is an urgent need for more effective biomarkers to enable early diagnosis and timely intervention.

Advancements in genomics, proteomics, and metabolomics have led to the identification of a diverse array of biomarkers in tumor tissues, serum, and urine. These biomarkers hold promise for applications in early diagnosis, prognosis, and the molecular characterization of predictive features, offering potential diagnostic, prognostic, and predictive utility. Diagnostic panels that amalgamate known biomarkers present a cost‐effective and time‐efficient avenue for the discovery of new markers. These panels can incorporate biomarkers from either the same or different biological specimens.

In conclusion, multi‐institutional collaborations are indispensable for ensuring adequate sample sizes when evaluating the efficacy of any new biomarker or diagnostic panel.

## AUTHOR CONTRIBUTIONS

**Jian Deng:** Project administration (equal); supervision (equal). **ShengYuan Tu:** Investigation (equal); visualization (lead); writing – original draft (equal); writing – review and editing (equal). **Lin Li:** Resources (equal); writing – original draft (equal); writing – review and editing (equal). **GangLi Li:** Project administration (equal); supervision (equal). **YinHui Zhang:** Conceptualization (lead); funding acquisition (equal); methodology (lead); resources (lead).

## FUNDING INFORMATION

This research received no external funding.

## CONFLICT OF INTEREST STATEMENT

The authors declare no conflict of interest.

## ETHICS STATEMENT

This is a literature review study, and no ethical approval is required according to the Ethics Committees of A.C.Camargo Cancer Center.

## Data Availability

Data sharing is not applicable to this article as no new data were created or analyzed in this study.
